# Physiology

**Published:** 1861-03

**Authors:** S. Weir Mitchell

**Affiliations:** Lecturer on Physiology in the Phila. Med. Association


### PHYSIOLOGY.

BY S. WEIR MITCHELL, M.D.,

LECTURER ON PHYSIOLOGY IN THE PHILA. MED. ASSOCIATION.

I.—*Zoospermes of the Frog; concerning their Vitality and the Transplantation of the Testicles of One Animal to Another.* Memoir which received a prize from the Academy at Brussels. By DR. PAOLO MANTEGAZZA.

Conclusions from the first part of the experiments upon the zoospermes of the frog:—

1. The zoospermes of the frog will live in any temperature from 13·75 degrees minus up to 43·75 degrees plus, (Centigrade.)

2. They will survive four consecutive freezings and thawings. They die after the fourth freezing only, after a long struggle with the cold.

3. They live for some time in a liquid sufficiently putrid for the development of infusoriæ.

4. They outlive the frog to which they belong a period varying from twelve hours to seven and a half days.

5. They live in a barometric vacuum.

6. They live excellently in the blood, in the mucus of the mouth, and in the urine of the frog

7. They subsist in human sperm, and in the water which frogs frequent.

8. They are killed by the gastric juice of the frog, phosphorated oil, alcohol, tincture of *coca*, acetic and sulphuric acids, by a solution of the ferrocyanate of potassium, and by chloroform.

9. Zoospermes live longer when left in the body than when the testicles are removed, the surrounding conditions of temperature and humidity being equal.

10. The individual constitution of the frog exercises a greater influence on the vitality of the zoosperme than the kind of death to which the frog is submitted.

For example: two frogs, equally robust, of the same age, killed at the same time by the destruction of the medulla spinalis, furnished zoospermes; those from one lived eighty-three hours and fifteen minutes; those from the other possessed more vitality, for they lived ninety-four hours and fifty-six minutes.

Three frogs, killed by submersion, presented these very different results as to the vitality of zoospermes:—

1 zoospermes lived 172 hours.
2 " " 157 "
3 " " 135 "

There is observed a still greater difference in the frogs which die spontaneously, for in these, upon autopsy, the zoospermes will almost always be found living.

Submersion in water which kills the healthy frog in nineteen hours, aids the vitality of the zoospermes, and those thus submerged resist death longer than others.

From all these observations we can form an idea of the tenacity of vitality in these histological elements. They resist the temperature of Senegal, and may be frozen and thawed four times in succession, without dying; up to a certain point they resist putrefaction, and have no need of atmospheric air for the maintenance of life.

The organs which constitute the microcosm of an animal mechanism are not merely portions closely connected by anatomical and physiological relations to the individual to which they belong, but they have also, by their structure and the purpose to which they are destined, an individuality which gives them a kind of automatic life. If the many facts already acquired by the conquests of science have not proved the physiological fact, it would be demonstrated with perfect clearness by the brilliant experiments of M. Ollier, upon the transplantation of periosteum. Still further, the experiments I am about to relate prove that an organ, very complex in its anatomical structure, and of great importance, in consequence of the function it fulfills in the organism, may be transplanted from one animal to another, and may continue to live in the new soil by its own peculiar life, for an indeterminate period.

Mantegazza now proceeds to give the details of fifty-seven experiments, in each of which he transplants a testicle of a frog to the body of another frog, male or female, either under the skin of the abdomen or thigh, and in almost all of them the transplantation was successful; that is, the testicle adhered to the tissues into which it was engrafted, and its zoospermes were found living at the expiration of various periods, the longest of which was 1650 hours, or more than two months and a quarter. The testicle, in many of these cases, became very vascular in its new location, and surrounded with a vascular envelope, through the medium of which it partook of the circulation of the blood of the animal into which it had been engrafted.

Signior Mantegazza says, in conclusion of this second part of his researches: These experiments prove that an organ as complex as the testicle may be transplanted from one individual to another, and live in the new organism for the space of seventy days. If it were possible to protract the life of frogs thus prepared still longer, by keeping them in ample and congenial reservoirs, we should doubtless observe phenomena still more important; because a gland would necessarily form for itself an excretory canal, and perhaps this gland, thus perfectly engrafted, might modify to a certain point the instincts of the animal into which it had been transplanted.

In the fourteenth and fifty-seventh experiments, it will be seen there was formed in each a true permeable tube or duct in the vicinity of the testicle; but, not having had the good fortune to see zoospermes issue from these ducts, I must, for the present, be contented to merely note the fact, leaving to future researches the choice of perfecting the observation.

Notwithstanding the close attention bestowed, I was not able to catch the instant in which the small tumor, which precedes the opening of the duct upon the surface of the skin, ruptured, in consequence of which I must, for the present, remain in doubt whether there was not an issue of true sperm at that moment, and whether the excretion was not arrested in consequence of the very bad conditions, as to nutrition, in which the frogs were placed.

To throw some light on this most obscure point, says Mantegazza, I will borrow, from Dr. Udikem, the fact observed by Wagner, who extirpated the testicle of a cock while it was yet a chicken, and re-engrafted it into the same bird, but in another part of the body, and found that when the cock became grown it had the crest and crow like other full-grown roosters. But Schwann, repeating the same experiment, found, on the other hand, that the transplanted testicle was by degrees absorbed, and the cock operated upon became a capon.

The transplanted testicle lives several days in the new organism, without attaching itself to any point, and I have often moved the testicle about under the skin for a period of two weeks, to break up its adhesions and prevent their formation, and thus preserve the testicle isolated under the teguments. In these cases, the organ lived by endosmosis. Somewhat later, the testicle flattened into a gelatinous stratum, which constitutes a sort of membraneous skin, and becomes fixed at some point of the new organism, getting into communication, by new blood-vessels, with the nutrient circulation of the frog. By its continued presence it may form a small excavation in the muscles under which it has been placed. When we have found the testicle adherent, we have not considered it necessary to resort to injection or to a fine dissection, to become convinced that the testicle had become a part of the new body, for the naked eye, or, at most, the eye aided by a loop, clearly perceived many blood-vessels surrounding the testicle and communicating with the organism.

The testicle lives in the female organism as well as the male, in the cavity of the belly as well as under the skin of the abdomen, or thigh, or back. (For the complete original essay, of which this is a mere abstract, see *Gaz. Med. Lomb.*, No. 26 et seq., July, 1860, translated in the *Pacific Med. and Surg. Journal*, No. 36, 1860.)

II.—*On the Excretion of Creatine and Creatinine in Health and Disease.*

Dr. Edward Schottin, of Dresden, has undertaken the laborious task of investigating the "Excretion of Creatine and Creatinine by the Urine and Effusions in the Healthy and Diseased Conditions of the Organism." Dr. Schottin has made use of his own urine, in order to determine the normal amount of creatine excreted. Under the use of a mixed diet, the quantity excreted could be seen only with the aid of the microscope: after strictly confining himself to vegetable food for thirty hours, no trace of creatinine was perceptible; while, after an almost exclusive use of animal food, he detected its presence to the amount of 0·086 of a milligramme. On taking half a milligramme of creatine, together with vegetable diet, no creatinine was observed.

The following cases of disease were investigated, with negative results:—

1. Tuberculosis of the lungs; the patient was much reduced in strength; there was high fever in the evening.
2. Tuberculosis of the lungs, with the formation of cavities; no fever.
3. Tuberculosis of the lungs and intestines; pleuritic exudation nine days before death. In the pleuritic and peritoneal exudations there was no creatine.
4. Granulated liver, with ascites and œdema of the feet.
5. Pneumonia, fourth day of the disease.
6. Bright's degeneration of the kidneys; general dropsy, without uræmic symptoms.
7. Progressive ataxy of the locomotive functions.
8. Paralysis of the arms, and weakness of the lower extremities, in consequence of chronic lead poisoning.
9. Chlorosis.
10. Spinal paralysis.

The cases in which creatine and creatinine appear as pathological secretions, Schottin then divides into two classes.

The first comprises those pathological changes in consequence of which the conversion of creatine and creatinine into bodies of a higher degree of oxydation is prevented; (as Bright's disease, with symptoms of uræmia.) The second class comprises, on the other hand, those cases in consequence of which the creatine is primarily increased by the degeneration of the striated muscles, (as typhus.)

As belonging to the first class, twenty-three cases of uræmia were investigated; in all of them a considerable increase of creatinine was observed. There were eleven cases of Bright's disease, with general dropsy; and at the various times at which they were examined, no increase of creatinine was found, and in most cases not a trace was observed.

The first series of cases furnished the following general results :—

1. The greater the disturbance of the secretion of the kidneys, the greater was the accumulation of creatine in the body.

2. The more urgent the symptoms of uræmia, the greater was the accumulation of creatine (creatinine?) in the blood.

3. The greater the secretion of the serous membranes, and the greater the œdema, the smaller was the accumulation of creatine (creatinine?) in the blood, and its elimination as creatinine in the urine.

Schottin remarks, further: "I have in these pages already repeatedly opposed the hypothesis of the conversion of urea in the blood into carbonate of ammonia, and of the consequent origin of uræmic symptoms. I am persuaded that the true reason of the occurrence of those symptoms is to be sought in this fact, viz., that by the obstructed secretion of urine in the kidneys, the general change of tissue is arrested in the brain and other parts of the body; and I have advanced the hypothesis that a lower grade of oxydation, thus determined, gives rise to the uræmic symptoms. I must still maintain my supposition, and I think I shall be able to offer satisfactory proof of it by means of the foregoing results. . . . . . A substance like creatinine, which has such an unusually strong alkaline reaction, and decomposes even the salts of ammonia, can act only as a poison upon the organism.

"Perhaps, too, the different observations upon the secretion of ammonia in uræmia and typhus have resulted only from the large amount of creatinine contained in the blood, in consequence of which ammonia was set free from its combinations; a view that is the more probable when we discover, further, how great an increase of creatinine takes place in the muscles and urine in typhus, with or without a disturbance in the secretion of the kidneys."

As belonging to the second class of the above-named diseases, Schottin examined twenty-two cases of abdominal typhus. The increase in the secretion of creatinine, which constantly takes place in these cases, begins generally in the middle of the second week of the disease. The quantity of creatinine in the urine rises to 0·2–0·3–0·6 of a milligramme.

The increase of the creatine contained in the muscles was also shown by Schottin. In the substance of the brain no such increase was found.

The author then notices a comprehensive pathological, anatomical, and histological investigation conducted by Professor Lenker, which has led to the result that in abdominal typhus a very extensive degeneration of the striated muscles is found, and this causes a partial destruction of the muscular fibres. "It seems" (page 437) "that the peculiar mode of the destruction of the muscle fibres determines the increase of creatine and creatinine."

With reference to the therapeutics, Schottin then shows that every increased formation of creatinine, especially when complicated with disturbance in the secretion of the kidneys, or produced by this disturbance, must occasion an abnormal alkalinity in the blood, and a formation of free ammonia, as far as possible. The chief indication, then, he thinks, is to oppose this alkaline condition, and he considers the use of those acids as especially called for which enter the blood as such, as the tannic and phosphoric acids.—(*Correspondenz Blatt des Verein für Gemeinschafteiche Arbeiten*, from the *Maryland and Virginia Medical Journal*, January, 1861.)

III.—*On Pulmonary Osmose; or Researches on Absorption and Exhalation by the Respiratory Organs.* By DR. LOUIS MANDL.

Mandl's extensive original essay being completed, we present our readers with a resumé of his experiments.

(*a*) Animals breathing in water cannot live when a more or less quantity of saccharine matter is dissolved therein. The substances experimented with include the true sugars, as cane sugar, beet sugar, glucose, sugar of milk, and the non-fermentable sweet principles, as glycerin, mannite. The rapidity with which these solutions act depends on the relative quantity of the sugar in solution, the quality of the sugar, and the species and condition of the animal.

(*b*) Experiments were made with a great number of different species of aquatic animals. They all lived much longer in solutions of cane sugar than in those of glycerin of the same strength. Thus, fishes, of the size of twelve to fifteen centimetres, (from five to six inches long,) perished in a solution with the tenth part of glycerin at the end of forty minutes, and in a solution of sugar only, at the end of four to five hours, all other circumstances being equal.

(*c*) Very numerous experiments have demonstrated that death can be attributed neither to poisoning, nor to chemical action on the blood, nor to fermentation, nor to the absence of air, nor to the viscosity, but that it is solely and entirely due to osmose (endosmose and exosmose) exercised by the saccharine solutions.

(*d*) This action goes on through all permeable membranes, and especially through those of the organs of respiration. The non-fermentable saccharine principles possess an osmotic power greater than that of the true sugars; and this explains the greater rapidity of their action. In infusoria this action goes on as in a bladder; osmose at once acts through the whole extent of their very thin integument and body; they are observed first to diminish, (exosmose,) then to swell up, (endosmose,) until they sometimes even burst; but the osmotic action going on over this whole extent is in all cases the cause of their rapid death. In animals of higher development, where the thickness of the teguments limits osmose principally to the gills, the blood is seen to thicken in the gills, and circulation to be arrested by exosmose of the liquid parts. The circulation in the lung of a frog can also be arrested over a limited space instantly with a drop of glycerin, and in a few minutes with simple syrup.

(*e*) Endosmometric experiments were made with animal membranes, (pericardium,) vegetable, (collodion,) and mineral, (unglazed porcelain,) to determine the solid constituents of the blood that pass into the saccharine liquid. It has been proven that the salts of the serum pass first, then albumen, then coloring matter.

(*f*) Development is equally arrested by saccharine solutions, as was proved by the experiments made with muscular tissue macerated in saccharine solutions, and those on fecundated eggs of fishes.

(*g*) Many physiological and pathological phenomena (to which we in this *summary* can, however, but cursorily refer) are referable to osmose exercised by saccharine solutions. Thus, the thirst excited by ingestion of sugar, which absorbs the water of the tissues with which it comes in contact; the antiseptic and preserving virtues of sugar, by the arrest of development of organisms; the digestive power of small quantities of sugar, which provoke the exosmose of the gastric juice; while large quantities introduced into the blood increase its osmotic power, which explains the use of sugar in the treatment of dropsies. The abundance of glucose in all the tissues of diabetic patients explains the constant thirst, the impossibility of any serious accumulation, and perhaps, also, by arrest of circulation, the gangrene sometimes observed in such patients. The use of glycerin as a local application is also founded on its great osmotic power. Accidental local tuberculosis, the diagnosis and treatment of which differ essentially from that of "diathesical" tuberculosis, is perhaps the result of sugar being carried into the lung; (but to this subject we will devote a new paragraph, and literally translate the author's language.)

(*h*) My studies on the etiology of tubercles (Mandl having previously demonstrated the fact that tubercle is not a specific product, but is produced from coagulation of secerned liquid substances of the blood) have long led me to think that, as to their origin, there are two distinct kinds of tubercles: the one depending on accident, the other on diathesis; the former only constitutes a local affection, the other, the symptoms of a constitutional disease. Experiments actually undertaken tended to produce local accidental tubercles, by the injection into the vesicles of the lung of osmogenetic substances, and particularly of saccharine matter.

While the limited number of my experiments imposes upon me great reserve, as yet, the results obtained give me hope of being able to prove, thus experimentally, the production of tubercles only localized in the lung, by accidental exudation (exosmose) of plastic matters, independent of all diathesis. These experiments will be published in due time. I will here only add, that they have led me back to the point of departure of the researches on osmose, and to the accidental production of tubercles in diabetes, where all the tissues are impregnated with glucose. These labors open a new field to therapeutics. They explain the cure, spontaneous or by art, frequently occurring in tuberculous affections of the lungs, not depending on diathesis, but purely local.—(*Arch. Gén. de Méd.*, July and August, 1860, from *Amer. Med. Monthly and N. Y. Review*, November, 1860.)

